# Lactate dehydrogenase A: A key player in carcinogenesis and potential target in cancer therapy

**DOI:** 10.1002/cam4.1820

**Published:** 2018-11-06

**Authors:** Yangbo Feng, Yanlu Xiong, Tianyun Qiao, Xiaofei Li, Lintao Jia, Yong Han

**Affiliations:** ^1^ Department of Thoracic Surgery, Tangdu Hospital Fourth Military Medical University Xi'an China; ^2^ State Key Laboratory of Cancer Biology, Department of Biochemistry and Molecular Biology Fourth Military Medical University Xi'an China

**Keywords:** cancer, glycolysis, lactate, LDHA, metabolism

## Abstract

Elevated glycolysis remains a universal and primary character of cancer metabolism, which deeply depends on dysregulated metabolic enzymes. Lactate dehydrogenase A (LDHA) facilitates glycolytic process by converting pyruvate to lactate. Numerous researches demonstrate LDHA has an aberrantly high expression in multiple cancers, which is associated with malignant progression. In this review, we summarized LDHA function in cancer research. First, we gave an introduction of structure, location, and basic function of LDHA. Following, we discussed the transcription and activation mode of LDHA. Further, we focused on the function of LDHA in cancer bio‐characteristics. Later, we discussed the clinical practice of LDHA in cancer prevention and treatment. What we discussed gives a precise insight into LDHA especially in cancer research, which will contribute to exploring cancer pathogenesis and its handling measures.

## 1 INTRODUCTION

Cancer cells possess distinct metabolism from non‐transformed cells, providing sufficient biomaterials and energy for infinite proliferation. Reprogrammed metabolism also contributes to metastasis, resisted cell death, and other malignant characters. Accelerated glycolysis constitutes an important aspect of cancer metabolism as reported by Warburg in 1956.^1^ High glycolytic activities supply precursors for biomolecules in cellular structure and processes. Importantly, lactate production in glycolysis contributes largely to malignant progression, like replenishing NAD^+^ for glycolysis, lowering pH for invasion, and triggering immune escape. Lactate dehydrogenase A (LDHA) converts pyruvate to lactate, and aberrant expression and activation of LDHA have been found closely related to diverse cancers.^2^, ^3^, ^4^ Therefore, LDHA has been regarded as a promising target for cancer prevention and treatment.

## 2 STRUCTURE, LOCATION, AND FUNCTION OF LDHA

Encoded by the *LDHA* gene, LDHA usually exists as tetramer (LDH‐5). LDHA contains 332 amino acids, forming a bilobal structure. The larger Rossmann domain provides sites for cofactors binding, while the smaller is for substrates.^5^, ^6^

The main function of LDHA is to convert pyruvate to lactate, and transform NADH to NAD^+^. When substrate and cofactor combine LDHA, activated site in the extended groove between two domains will be enclosed in rid of dissociated solvent. Subsequently, Arg 105 in activated circles will grip adhered pyruvate, while the hydrogen anion will transfer from nicotinamide ring of NADH to oxygen atom in carbonyl of pyruvate.^6^, ^7^

LDHA is mainly located in the cytoplasm, but LDHA has also been found in the mitochondria and nucleus.^8^, ^9^, ^10^ Outside the nucleus, LDHA play a critical role in glycolysis, while in the nucleus, LDHA function as a single‐stranded DNA‐binding protein (SSB), likely participating in DNA duplication and transcription.

## 3 REGULATION MODES OF LDHA

**Figure 1:**
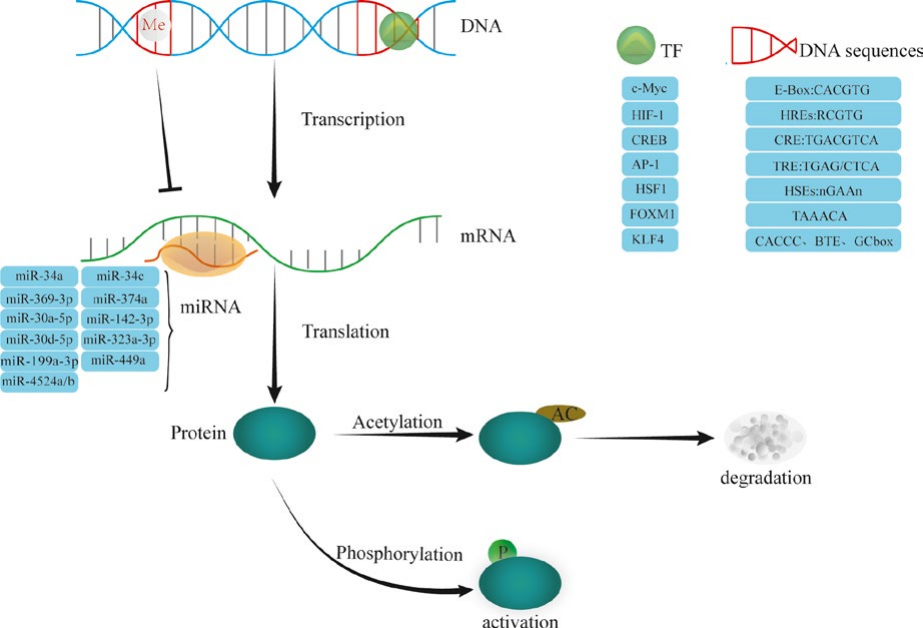
Regulation modes of LDHA. LDHA can be regulated in almost every step of gene expression. Methylation modification could repress LDHA transcription; various transcription factors can function at respective elements in LDHA promoter to activate or curb LDHA transcription; many kinds of microRNAs can bind to mRNA of LDHA to hinder its translation or induce degradation; phosphorylation can activate LDHA, while acetylation triggers its degradation by proteasome. AP‐1, activator protein‐1; CREB, cAMP response element‐binding protein; FOXM1, forkhead box protein M1; HIF‐1, hypoxia‐inducible factor‐1; HSF1, heat‐shock factor 1; KLF4, Kruppel‐like factor 4; TF, transcription factor

In many cancers, LDHA has a high expression profile and activated status, attributed to diverse mechanisms involving almost every step of gene expression regulation (Figure 1).

### 3.1 Epigenetic modification

*LDHA* gene is located in the shorter arm of chromosome 11, a hot spot for methylation. Many researches have proved that DNA methylation in the promoter inhibited LDHA expression. Chesnelong et al found glioma with mutant isocitrate dehydrogenase (IDH) had a low‐level LDHA compared with IDH wild‐type, and the molecular underpinning might be that mutant IDH causes higher methylation in *LDHA* promoter.^11^ Besides, Maekawa et al reported retinoblastoma cell line NCC‐RbC‐51 hardly express LDHA, which, however, would be restored by demethylating agent 5‐aza‐2′‐deoxycytidine.^12^ These all suggest that methylation modification plays a vital part in LDHA activation mechanisms.

### 3.2 Transcriptional regulation

Promoter region of *LDHA* contains multiple elements for diverse transcription factors binding; thus, LDHA could be regulated by numerous transcription factors.

#### 3.2.1 c‐Myc

Transcription factors c‐Myc functions as an essential oncogene in numerous cancers.^13^ c‐Myc could associate with myc‐associated factor X (MAX) to form heterodimer, which binds to E‐box (5′‐CACGTG‐3′) in the promoter of LDHA, thus transactivating LDHA expression.^14^ In pancreatic cancer, c‐Myc expression was positively related to LDHA, and knockdown of c‐Myc could inhibit LDHA expression, impairing lactate production and glucose consumption.^15^ Intriguingly, Zhang et al^16^ found that inhibition of LDHA increased c‐Myc expression, suggesting that LDHA had a negative feedback on c‐Myc expression.

#### 3.2.2 HIF‐1

Hypoxia‐inducible factor‐1 (HIF‐1) is a heterodimer containing α and β subunits. When in hypoxia, stabilized HIF‐1α enters into the nucleus to combine HIF‐1β, and then, such complex binds to hypoxia‐responsive elements (HRE) to transactivate targeted genes. In non–small‐cell lung cancer (NSCLC), Koukourakis et al^17^ proved that LDHA was positively related to HIF‐1α. Later, Semenza et al^18^ found HRE existed in the promoter region of LDHA and demonstrated that HIF‐1 could occupy HRE (5′‐RCGTG‐3′) to transactivate LDHA expression. In addition, c‐Myc and HIF‐1 could collaborate to activate LDHA transcription in various cancer cells.^19^

#### 3.2.3 CREB

Once phosphorylated by the activated cAMP‐PKA signaling pathway, the transcription factor cAMP response element–binding protein (CREB) could engage the cAMP response element (CRE) containing 5′‐TGACGTCA‐3′ sequence, thereby initiating transcription of target genes. The promoter region of LDHA gene also has a highly conserved CRE sequence; thus, LDHA could be regulated by CREB as well.^20^

#### 3.2.4 AP‐1

12‐O‐Tetradecanoylphorbol‐13‐acetate (TPA) could activate protein kinase C (PKC) signaling pathway, consequently phosphorylating transcription factor activator protein‐1 (AP‐1), which binds to the TPA‐responsive element (TRE) to trigger target gene expression. The promoter region of LDHA gene has a TRE (5′‐TGAG/CTCA‐3′); thus, LDHA could be transcriptionally regulated by AP‐1.^21^, ^22^

#### 3.2.5 HSF1

Heat‐shock factor 1 (HSF1), a common transcription factor, regulates heat‐shock protein (HSP) to restore protein homeostasis. In cellular stress, HSF1 forms a transcriptionally active trimer; meanwhile, HSF1 exporting into the cytoplasm is inhibited, and aggregated HSF1 in the nucleus could bind to the heat‐shock elements (HSEs) (nGAAn pentamer inverted repeats) of the heat‐shock protein gene. HSF1 can also function as a transcription factor to regulate the expression of LDHA. Researchers showed that ErbB2 could increase HSF1, which is enriched in promoter region of LDHA, enhancing LDHA expression.^23^

#### 3.2.6 FOXM1

Forkhead box protein M1 (FOXM1) belongs to Forkhead transcriptional superfamily, recognizing 5′‐TAAACA‐3′ tandem repeat sequences in the promoter region to mediate the transcription of targeted genes like those involved in cell cycle progression.^24^ FOXM1 has been reported to regulate LDHA expression as well. Cui et al^25^ found FOXM1 level was positively associated with LDHA in pancreatic cancer. Further, Jiang et al^26^ demonstrated FOXM1 bound to the promoter of LDHA and promoted its transcription in gastric cancer.

#### 3.2.7 KLF4

Kruppel‐like factor 4 (KLF4) is a zinc‐finger transcription factor, mainly expressed in terminal differentiation of epithelial cells.^27^, ^28^ KLF4 transferred into the nucleus exerts transcriptional regulation by binding to the GC box, 5′‐CACCC‐3′ sequence, or basic transcription element (BTE) of the promoter region within target genes.^29^, ^30^ KLF4 can also regulate the expression of LDHA. Shi et al^31^ found KLF4 was negatively related with LDHA level, and KLF4 bound to −371 to −367 bp or −1310 to −1306 bp promoter region of LDHA.

### 3.3 Posttranscriptional regulation

MicroRNA (miRNA), a kind of small non‐coding RNA, could inhibit translation or promote degradation of targets via combining to the specific region of 3′‐untranslated region (3′‐UTR) in targeted mRNA.^32^, ^33^ Until now, several miRNAs like miR‐34a, miR‐34c, miR‐369‐3p, miR‐374a, miR‐30a‐5p, miR‐142‐3p, miR‐30d‐5p, miR‐323a‐3p, miR‑199a‑3p, miR‐449a, and miR‐4524a/b have been found to target the mRNA of LDHA.^34^, ^35^, ^36^, ^37^, ^38^, ^39^, ^40^, ^41^, ^42^, ^43^ In a recent study on colorectal cancer, miR‐34a, miR‐34c, miR‐369‐3p, miR‐374a, and miR‐4524a/b were established to directly inhibit LDHA.^34^ Kaller et al^32^ proved miR‐34a targeting of LDHA via luciferase reporter assay. In advanced colon cancer, miR‐34a was significantly down‐regulated in 5‐fluorouracil‐resistant cancer tissues, while the expression of LDHA was abnormally increased. The expression of LDHA was positively correlated with 5‐fluorouracil resistance; LDHA could be suppressed by introduction of miR‐34a, which could restore the sensitivity of advanced colon cancer to 5‐fluorouracil.^44^ Besides, miR‐34a could indirectly inhibit LDHA expression by regulating cytokines.^45^ Thus, these miRNAs play a significant part in negative regulation of LDHA via posttranscriptional modification.

### 3.4 Posttranslational modification

LDHA could also be modulated by posttranslational modification as exemplified by phosphorylation and acetylation in specific amino acids.

Phosphorylation significantly increased enzymatic activity of LDHA, which is associated with cancer progression. In breast cancer, Jin et al^46^ revealed that Y10 phosphorylation elicited LDHA activation, promoting cancer cell invasion and enhancing anoikis resistance. Also in colorectal cancer, Ji et al^47^ found that human coilin–interacting nuclear ATPase protein (hCINAP) expression was positively correlated with the level of Y10 phosphorylated LDHA. The molecular mechanism is that hCINAP binds to the C‐terminal region of LDHA and depends on its own adenylate kinase activity to promote phosphorylation of LDHA catalyzed by fibroblast growth factor receptor 1 (FGFR1) at Y10.^47^ Furthermore, Fan et al^48^ found that direct phosphorylation of LDHA at Y10 and Y83 significantly enhanced LDHA tetramer formation and cofactor binding, resulting in a significant increase in lactate dehydrogenase activity.

In addition, lysine acetyl also participates in the regulation of LDHA activity. In human pancreatic cancer, a decrease in acetylated levels of LDHA K5 leads to activation of LDHA enzyme activity and inhibition of LDHA degradation.^49^ Zhao et al^50^ showed that in pancreatic cancer LDHA could be deacetylated at K5 by the deacetylase sirtuin 2 (SIRT2). In addition, they found that acetylation on LDHA K5 leads to degradation of LDHA, the underlying mechanism is that the K5‐acetylated LDHA is recognized by the heat‐shock cognate protein 70 (HSC70) and delivered to lysosomes for degradation.^50^

## 4 LDHA IN CANCER BIOLOGY

**Figure 2:**
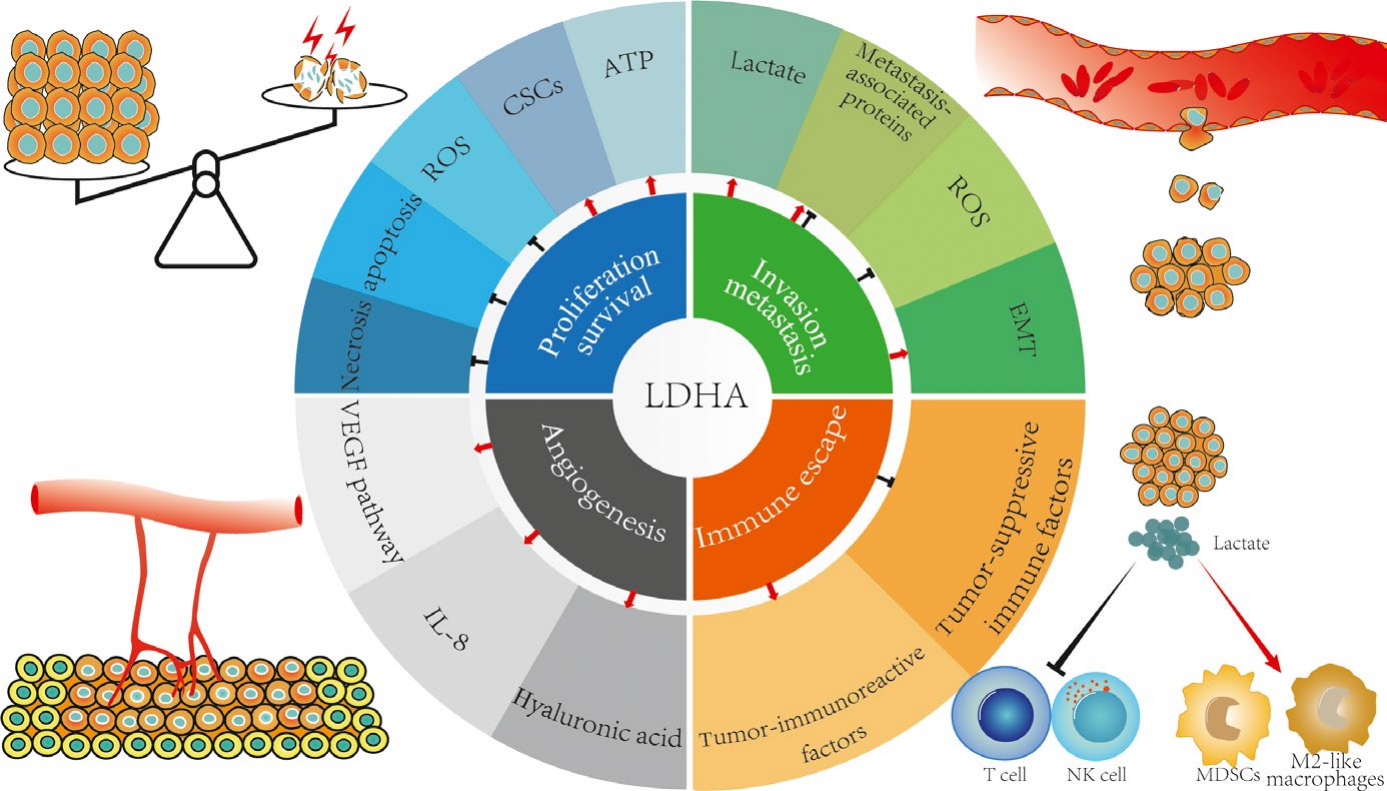
LDHA plays critical roles in hallmarks of cancer. LDHA is closely related to malignant bio‐characteristics of cancer via various mechanisms. LDHA can promote cancer cell proliferation and maintain cell survival; LDHA helps enhance cancer cell invasion and metastasis; LDHA can also trigger angiogenesis; LDHA can assist cancer cells in immune escape. ATP, adenosine triphosphate; CSCs, cancer stem cells; EMT, epithelial‐to‐mesenchymal; IL‐8, interleukin‐8; MDSCs, myeloid‐derived suppressor cells; ROS, reactive oxygen species; VEGF, vascular endothelial growth factor

In cancers, enhanced LDHA promotes diverse malignant bio‐characteristics. (Figure 2).

### 4.1 Proliferation and survival

Unlimited proliferation and resisted cell death are both hallmarks of cancer, and LDHA contributes greatly to cancer proliferation and survival. Yao et al^51^ found that LDHA expression was up‐regulated in clinical samples of esophageal squamous cell carcinoma, and LDHA knockdown could inhibit cell growth and migration in vitro and impair tumorigenesis in vivo. Miao et al's^52^ study on human hepatocellular carcinoma (HCC) also showed that LDHA‐knockdown cells underwent apoptosis. Small molecule inhibitor of human LDHA effectively inhibited tumor growth in human B‐lymphoma and pancreatic cancer xenograft models.^3^ A study by Dorneburg et al^53^ on neuroblastoma illustrated that knockout of LDHA suppressed tumor growth and tumorigenicity. Additionally, in K‐RAS‐induced NSCLC mouse model, Xie et al^54^ demonstrated that LDHA deletion could intensely reduce tumorigenicity.

There have been several explanations for the molecular mechanisms underlying the division and survival of cells with hyperactivity of LDHA. First, LDHA can supply sufficient energy for cancer cells. Fantin et al^4^ found that under normoxic conditions, the proliferation rate of the cells decreased after downregulating LDHA, and under hypoxic conditions (0.5% oxygen), the growth of tumor cells with LDHA deficiency was also seriously impaired. By measuring adenosine triphosphate (ATP) levels in LDHA‐defective tumor cells under normoxic and hypoxic conditions, they found that tumor cells with reduced LDHA activity could not maintain high ATP levels, which probably contributed to the retardation of cell proliferation under normoxic or hypoxic conditions. Such energy providing efficiency relies on metabolic phenotype of cancer. Cancer cells dependent on glutamine decomposition and fatty acid synthesis are not affected by LDHA inhibitors, because these cells are more dependent on mitochondrial function to produce ATP once the production of lactic acid is inhibited. However, cell lines dependent on the pentose phosphate pathway and glycolysis are dramatically affected by LDHA inhibitors.^55^, ^56^

Second, LDHA may be involved in promoting cancer stem cells (CSCs) phenotype. Zhang et al^16^ found that LDHA was significantly associated with octamer‐binding transcription factor 4 (Oct‐4), which functions critically in self‐renewal of embryonic stem cells. They found that knockdown of LDHA could reduce Oct‐4 expression and tumorigenicity in vitro and in vivo.^16^

Additionally, LDHA indirectly promotes tumor survival by protecting the tumor from reactive oxygen species (ROS) damage. Mitochondrial ROS is usually elevated because inhibition of LDHA forces cancer cells to produce ATP through oxidative phosphorylation.^3^, ^4^, ^57^ Studies using in vitro and in vivo xenograft mouse models found that treatment of LDHA‐inhibited cells with N‐acetyl‐L‐cysteine (NAC) prevented or partially prevented ROS‐induced apoptosis.^2^ Accordingly, Sheng et al^2^ proposed a model of LDHA‐induced apoptosis in human hepatoma cells: LDHA inhibition induced the production of ROS and released cytoplasmic Ca^2+^, which decreased mitochondrial inner membrane potential (ΔΨm), activated caspase‐9 and caspase‐3, and eventually induced apoptosis.

Moreover, LDHA may also directly inhibit apoptosis. Zhuang et al's^58^ immunohistochemical study of melanoma revealed that LDHA was strongly correlated with the expression of the antiapoptotic proteins myeloid cell leukemia‐1 (Mcl‐1) and B‐cell lymphoma‐extra large (Bcl‐XL). LDHA knockdown increased poly (ADP‐ribose) polymerase (PARP) cleavage and decreased X‐linked inhibitor of apoptosis protein (XIAP), B‐cell lymphoma 2 (Bcl‐2), and Bcl‐XL expression, leading to decreased the tumorigenicity of a pancreatic cell line, BxPC‐3, in a xenograft model.^59^ In xenografts of breast cancer cell lines, LDHA knockout was also found to have elevated the levels of Bcl‐2‐associated X protein (Bax), cleaved PARP, cleaved caspase‐9, cytoplasmic cytochrome C, and superoxide anion, while Bcl‐2 expression and mitochondrial membrane potential were reduced.^60^

Finally, overexpression of LDHA can also promote tumor growth by preventing necrosis in hypoxic environment. Lewis et al^61^ pointed out that tumors overexpressing LDHA and Rcl displayed little necrotic region compared to tumors overexpressing Rcl and vascular endothelial growth factor (VEGF). This indicates that elevated LDHA expression protected central tumor cells from hypoxia‐induced necrosis.^61^

### 4.2 Invasion and metastasis

Invasion and migration are prominent characteristics for malignant progression. LDHA significantly affects the invasion and migration of malignant cells. In Miao et al's^52^ study, LDHA‐inhibited hepatoma cells exhibited approximately half reduction in motility compared to control cells. Koukourakis et al^62^ found a positive correlation between LDHA expression and distant metastasis of colorectal cancer. A study by Xie et al^63^ on hereditary leiomyomatosis and renal cell carcinoma (HLRCC) syndromes reported that fumarase (FH)‐deficient cells had a strong invasive potential, while inhibition of LDHA activity inhibits the mobility of cells.

The most common mechanism by which LDHA regulates cell migration and invasion is ascribed to lactate output. Lactic acid levels are related to the incidence of distant metastases.^64^, ^65^ High concentrations of lactic acid are associated with high rates of early distant metastases in cancer.^66^ Exogenous lactic acid can increase tumor cell motility and random migration of different cancer cell lines.^67^ The effect of lactic acid on metastasis mainly includes activation of matrix metalloproteinases (MMPs) and cathepsin by acidic microenvironment, up‐regulation of VEGF, HIF‐1α, and transforming growth factor‐β2 (TGF‐β2), as well as direct enhancement of cell migrative ability.^66^, ^68^, ^69^, ^70^, ^71^

Second, LDHA can regulate metastasis‐associated proteins. Sheng et al^2^ showed that knockdown of LDHA could reduce the expression of focal adhesion kinase (FAK), matrix metalloproteinase‐2 (MMP‐2) and VEGF, and increase the expression of E‐cadherin. These results suggest that LDHA may affect tumor migration and invasion through the regulation of key players in these cellular processes, for example, inducing degradation of extracellular matrix via stimulating MMP‐2 production, promoting metastatic vasculogenesis through up‐regulation of VEGF, and inhibiting cell adhesion by inhibiting E‐cadherin.

Third, LDHA regulation of invasion and metastasis also involves the production of ROS. In melanoma, Arseneault et al^72^ found that LDHA inhibition resulted in mitochondrial ROS accumulation, which may alter the structure of tropomyosin via oxidation. Thus, altered tropomyosin could impair its ability to promote actin remodeling, leading to decreased melanoma migration. However, after treatment with antioxidants NAC, migration inhibition and cytoskeletal defects caused by LDHA knockdown could be partially rescued.^72^

Furthermore, activating epithelial‐to‐mesenchymal transition (EMT) also underlies LDHA contribution to cancer metastasis.^73^ In bladder cancer, Jiang et al^73^ found that knockdown of LDHA prevented invasion of cancer cells, accompanied by decreased Snail, N‐cadherin, fibronectin, and vimentin expression but increased E‐cadherin expression.

### 4.3 Angiogenesis

Angiogenesis is also a core character of malignancy, which is mediated primarily by extracellular factors such as VEGF and interleukin‐8 (IL‐8). Diverse studies have shown that LDHA could regulate tumor angiogenesis. Koukourakis et al found that LDHA expression was positively associated with activation of the VEGF signaling pathway.^62^, ^74^, ^75^, ^76^ In studies on endometrial cancer, it was confirmed that LDHA expression was significantly related with the activation of vascular endothelial growth factor receptor 2 (VEGFR2) phosphorylation.^74^ High expression of LDHA was also strongly associated with VEGF expression in non‐small‐cell lung cancer.^17^ Activated VEGF/VEGFR2 signaling could remarkably promote angiogenesis. At the same time, clinical trials have shown that cancer patients with enhanced LDHA expression could significantly benefit from anti‐angiogenic therapy, suggesting that LDHA may accelerate tumor progression by facilitating angiogenesis.^76^, ^77^

Regulation of angiogenesis by LDHA is mainly dependent on the production of lactic acid. Acidification in the microenvironment promotes the production of IL‐8 and VEGF.^78^ Beckert et al^79^ confirmed that lactic acid stimulated endothelial cells to produce VEGF and induced neovascularization. In a wound‐healing mouse model, subcutaneously sustained local lactate release could promote angiogenesis and accelerate wound healing.^80^ Vegran et al^81^ also found that uptake of lactic acid by vascular endothelial cells triggered the phosphorylation/degradation of IκBα, activated nuclear factor‐kappa B (NF‐κB), promoted IL‐8 expression, and subsequently accelerated angiogenesis and tumor growth. In addition, lactic acid can also promote angiogenesis by increasing the production of hyaluronic acid.^82^, ^83^

### 4.4 Immune escape

Cancer possesses a flexible strategy to escape from immune surveillance. Highly expressed LDHA mediates tumor immune escape by inhibiting immune killing and promoting immunosuppression. The expression of LDHA in human melanoma is negatively correlated with the T‐cell activation markers such as granzyme K (GZMK) and CD25.^84^ The number of cytotoxic effector cells in tumors with low expression of LDHA was high compared to those with abundant LDHA.^84^ Husain et al^85^ observed a decrease in the number of myeloid‐derived suppressor cells (MDSCs) in the spleen of tumor‐bearing mice after depletion of LDHA. Additionally, Seth et al^86^ found in K‐Ras‐induced cancer mouse model that LDHA deletion diminished PD‐L1+ tumor cells but elevated CD3+ and CD8+ T cells, suggesting significant immune activation in these mice.

The main mechanism of LDHA suppression of immunity is the increase in lactate output, which could impair function of tumor‐suppressive immune factors. First, the acidic microenvironment caused by lactate inhibits the production of interferon‐γ (IFN‐γ) by immunocompetent cells. Brand et al^84^ found that high expression of LDHA in melanoma resulted in accumulation of lactic acid, and the acidic environment downregulated nuclear factor of activated T cells (NFAT) in T and NK cells, which in turn led to a decrease in IFN‐γ production and attenuated the function of tumor‐infiltrating T cells and NK cells, triggering tumor immunosuppression. Acidic conditions can also inhibit the activity of calcineurin, which in turn disturbs nuclear translocation of NFAT, and impair antitumor immunity.^87^ Lactic acid also inhibits the glycolytic function of glyceraldehyde phosphate dehydrogenase (GAPDH), allowing it to bind to IFN‐γ mRNA, thus preventing the translation of IFN‐γ.^88^, ^89^ In addition, it has also been reported that lactic acid could directly cause apoptosis of T cells and NK cells.^84^

The accumulation of lactic acid upregulates the levels of tumor‐immunoreactive factors. Shime et al showed that lactic acid promoted the expression and secretion of interleukin‐23 (IL‐23), which impeded infiltration of CD8+ T cells in tumor microenvironment.^90^, ^91^ Colegio et al showed that lactic acid upregulated HIF‐1α to facilitate the development of M2‐like macrophages, a subset of macrophages known to promote cancer progression.^92^, ^93^ Besides, MDSCs could prohibit function of T cells and NK cells to suppress immune response.^94^ LDHA‐induced lactate accumulation could also increase the number of MDSCs in immune escape of tumors.^85^

## 5 LDHA AS A BIOMARKER AND THERPEUTIC TARGET FOR CANCER

### 5.1 LDHA as a biomarker for cancer diagnosis and prognosis

Lactate dehydrogenase (LDH) is present in cells. When cells are damaged, they are released into the bloodstream. Therefore, LDH levels in the blood are usually used as indicators of tissue damage. Serum LDH levels also have momentous significance in cancer diagnosis due to tissue destruction caused by tumor growth.^52^ In general, serum LDH is usually elevated in hematopoietic malignancies such as Hodgkin's lymphoma (HL) and non‐Hodgkin's lymphoma (NHL).^95^, ^96^, ^97^, ^98^ Colgan et al^99^ found that surgical removal of the primary tumor resulted in a dramatic decrease in serum LDH levels within first week after surgery. Tumor metastasis can lead to elevated LDH levels, suggesting that LDH may be a potential diagnostic marker for cancer.

Additionally, LDH could serve as an indicator of the prognosis of malignancy. LDHA is a strong predictor of survival in patients with aggressive lymphoma as one of the risk factors in the International Prognostic Index (IPI). Jin et al^100^ performed a retrospective analysis of the relationship between pre‐ and post‐treatment serum LDH levels and the clinical response and survival outcomes of patients with metastatic nasopharyngeal carcinoma. The results showed that patients with elevated pre‐treatment serum LDH levels had a worse survival rate compared with those displaying normal pre‐treatment serum LDH. Compared with those with normal post‐treatment serum LDH levels, the survival rate of patients with elevated serum LDH was also significantly lowered.^100^ Due to no clinical differences in the specific subtypes of LDH, the separate role of serum LDHA in diagnosis and prognostic prediction still needs further investigation.

In addition to serum LDH, LDHA in tissues can often be used as a biological indicator of diagnostic and malignant characteristics of tumors. Kayser et al^101^ found that almost 90% of NSCLC patients were positive for LDHA, while all non‐tumor lung tissues showed negative LDHA expression. In addition, the staining intensity of LDHA was found to be highly correlated with the histological type and lymph node metastasis of lung cancer, indicating that the tissue level of LDHA had prognostic value in NSCLC.^101^ Up‐regulation of LDHA levels in tumor tissues can be observed in pancreatic cancer and esophageal squamous cell carcinoma (ESCC), which was associated with metastasis, tumor stage, tumor recurrence, and patient survival.^25^, ^51^

However, LDHA expression in cancer tissues is not completely consistent with serum LDH levels, which may indicate that tumor LDHA expression and serum LDH levels are two independent predictors of tumors.^102^ Koukourakis et al^62^ found that patients with low levels of LDHA in tumor tissues often had low levels of serum LDH, but only 29% of patients with high LDHA expression had elevated serum LDH levels, and 71% of patients with high LDHA expression levels showed normal serum levels. Dong et al^102^ also found that the expression level of LDHA in triple‐negative breast cancer tissues was not completely consistent with the serum LDH level. Koukourakis et al^62^ proposed that the level of serum LDH in normal individuals was substantially different, masking the increase in LDHA caused by tumorigenesis.^62^ Thus, serum LDH and tissue LDHA levels can complement each other and exert a combined role in diagnosis of cancer.^76^

### 5.2 LDHA as a target for cancer treatment

As is shown above, numerous tumors exhibit high LDHA expression, which contributes to malignant bio‐characteristics.^103^, ^104^, ^105^ Silencing LDHA expression in tumor models inhibits cell proliferation, migration, and tumor growth.^51^, ^59^, ^72^, ^106^ However, it is rarely harmful to normal cells.^57^, ^107^ Additionally, individuals lacking LDHA subunits only develop muscle rigidity and sudden myoglobinuria after strenuous exercise.^108^, ^109^ So, high anticancer efficacy along with safe therapeutic window enables LDHA to be a potential antitumor target. Based on the functional mechanisms, LDHA inhibitors could be divided into the following categories (Table 1).

**Table 1:** Diverse drugs that target LDHA

Mechanism of action	LDHA inhibitor	Cancer	Clinical trials	Drawbacks	References
Pyruvate‐competitive	Oxamate	Gastric cancer; cervical cancer	No	Limited membrane permeability	110, 111
NADH‐competitive	Gossypol	Melanoma; lung cancer; breast cancer; cervical cancer; leukemia; glioma; adrenal cancer	Yes	Non‐specific toxicity	112, 113, 114, 115, 116, 117, 118
	FX11	B‐lymphoma; pancreatic cancer	No	Highly reactive catechol portion of this molecule	3, 5, 120
	Quinoline 3‐sulfonamides	Hepatocellular carcinoma	No	Low clearance in the body and incompatible with oral bioavailability	55
Pyruvate and NADH competitive	NHI	Pancreatic ductal adenocarcinoma; cervical cancer	Unknown	Unknown	9, 120, 121
Binding the free enzyme	Galloflavin	Breast cancer; hepatocellular carcinoma	Unknown	Unknown	122, 123, 124

#### 5.2.1 Pyruvate‐competitive LDHA inhibitor

As an analogue of pyruvate, oxamate inhibits LDHA via competition with substrates, and its effectiveness has been extensively validated in vitro. In a study on gastric cancer, the human gastric cancer cell lines SGC7901 and BGC823 had higher LDH activity and more lactic acid levels than the immortalized gastric mucosa epithelial cell line GES‐1. Oxamate significantly inhibited the proliferation and lactic acid production of SGC‐7901 and BGC‐823 cells in a dose‐ and time‐dependent manner, but showed less effect on GES‐1 cells.^110^ Similarly, after treatment with oxamate, the growth and lactic acid production of HeLa cells were also inhibited.^111^ Unfortunately, due to limited cell membrane permeability, the effective dose of oxamate in cultured cancer cells is too high for in vivo administration.

#### 5.2.2 NADH‐competitive LDHA inhibitors

Gossypol, a natural phenol derived from cotton plants, inhibits LDHA by competing with NADH.^112^ Gossypol showed dose‐dependent cytotoxic activity in diverse cancer cells, including melanoma (SK‐mel‐19, SK‐mel‐28), small‐cell lung cancer (H69), breast cancer (Walker), cervical cancer (Sihas), myelogenous leukemia (K562), and glioma (HS683, U373, U87, and U138).^113^, ^114^ Meanwhile, gossypol shows satisfactory anticancer efficiency in vivo. Coyle et al^114^ used a nude mouse xenograft model to test the cytotoxicity of Gossypol on BRW (a cell line established from a patient with a primitive neuroectodermal tumor). After oral administration of gossypol by gavage, the average tumor weight was reduced by more than 50%.^114^ Furthermore, several clinical trials have demonstrated promising tumoricidal potential of gossypol. In metastatic adrenal cancer, Flack et al^115^ found that after oral administration of Gossypol, the tumor volumes of patients was reduced by half, while the only serious side effect was intestinal obstruction. Likely, oral administration of Gossypol also benefits malignant glioma patients who had a relapse after radiotherapy.^116^ In a phase I/II clinical study, 20 women with refractory metastatic breast cancer received oral treatment with Gossypol. The results showed that Gossypol treatment was safe and effective in decreasing serum tumor markers.^117^

Nevertheless, gossypol could interact with different cellular components involved in several biological functions, resulting in non‐specific toxicity of this compound. So further drug‐making attempts have produced frustrating results.^112^, ^118^ Therefore, many gossypol analogs are being designed and under evaluation for their safety and anticancer efficacy.

FX11 (11f; [3‐dihydroxy6‐methyl‐7‐(phenylmethyl)‐4‐propylnaphthalene‐1‐carboxylic acid]) is also a competitive LDHA inhibitor through competing with NADH. The human B‐lymphoma P493 cell line treated with FX11 showed abundant cell death. FX11 also inhibits tumor progression and induces oxidative stress and necrosis in human lymphoma and pancreatic cancer xenograft models.^3^

However, further studies questioned the effectiveness of FX11 since some of the observed effects may not be attributed to LDH inhibition, but rather to the reactive nature of the catechol groups of the molecule.^5^, ^119^ Therefore, although FX11 exhibits certain therapeutic potential, the highly reactive catechol portion of this molecule makes it unsuitable as a candidate drug for further development.

Quinoline 3‐sulfonamides are NADH‐competitive LDH inhibitors with higher selectivity for LDHA than lactate dehydrogenase B (LDHB). After treatment with quinoline 3‐sulfonamides, hepatocellular carcinoma Snu398 cells showed increased oxygen consumption, inhibited lactic acid production and cell proliferation, and promoted apoptosis. However, due to low clearance in the body and incompatible with oral bioavailability, it cannot be used in vivo.^55^

#### 5.2.3 Pyruvate and NADH‐competitive LDHA inhibitors

N‐hydroxyindoles (NHI), a distinguished class of LDH inhibitors that compete with substrates (pyruvate) and cofactors (NADH), are more specific for LDHA than LDHB. Cellular assays have confirmed that they can impede cancer cell proliferation. For example, when treated with NHI compounds, pancreatic ductal adenocarcinoma cells exhibited decreased growth and invasiveness especially under hypoxic conditions. NHI could inhibit cervical cancer HeLa cells as well.^120^ In addition, NHI compounds can synergize with gemcitabine to exert antitumor effects.^121^ Further exploration of biological properties of NHI will help better assess its therapeutic potential for various cancers.^9^

#### 5.2.4 Free enzyme‐binding inhibitor

Galloflavin (GF), a synthetic drug with good cell permeability, inhibits LDHA by preferentially binding to free enzymes without competing with substrates or cofactors.^122^ It is harmless to mitochondrial respiration, and showed minimal effects on normal cellular metabolism. In breast cancer cell lines, GF inhibits cancer cell proliferation by blocking glycolysis and ATP production, and induces apoptosis.^123^ GF inhibits aerobic glycolysis and trigger cell death in hepatocellular carcinoma cell line PLC/PRF/5.^122^ GF is less cytotoxic to normal cells such as human lymphocytes and lymphoblasts.^123^ A recent study found that GF interferes with LDHA/ssDNA interactions and blocks RNA synthesis in vitro, suggesting the complicated mechanism underlying its toxicity to malignant cells.^124^

#### 5.2.5 Other unknown mechanisms

Genetech Corporation synthesizes another LDHA inhibitor, GNE‐140, a piperidine derivative, which has been shown to effectively inhibit the proliferation of MiaPaCa‐2 pancreatic cancer cells. In addition, a natural extract with the ability to inhibit LDHA, named gall, has also been found to be very effective in cancer treatment.^125^

Apart from being applied alone, studies have shown that LDHA inhibitors in combination with other agents exhibited dramatic therapeutic capacity. Miskimins et al^126^ found that oxamate and phenformin synergistically exerted anticancer effects by simultaneously inhibiting complex I in mitochondria and LDH in cytosol. By combining with the NAD^+^synthetic inhibitor FK866, FX11 induces lymphoma regression remarkably.^3^ In pancreatic ductal adenocarcinoma cell lines, the combination of NHI‐1 and NHI‐2 with gemcitabine enhances the anti‐proliferative and anti‐invasive activity of chemotherapeutic drugs both under normoxic or hypoxic conditions.^121^ In addition, NHI‐2 and a redox‐dependent bioreductive anticancer prodrug, EO9, synergistically induce the death of p53 wild‐type cancer cells.^127^

## 6 CONCLUSIONS AND PERSPECTIVE

The unique metabolic pattern of tumor cells significantly promotes its malignant biological characteristics, and abnormally regulated metabolic enzymes are the basis of its metabolic reprogramming. In many malignancies, LDHA may be abnormally overexpressed due to activation of LDHA upstream regulatory mechanisms by cancer‐driving mutations. Up‐regulated LDHA promotes the malignant progression of tumors by increasing lactic acid production, accelerating glycolysis, modulating reactive oxygen species production, and regulating numerous cancer‐related proteins. At the same time, clinical trials using LDHA as a target for diagnosis and treatment have also yielded encouraging results.

However, there are still many problems that remain to be resolved. First of all, it is unclear whether and how LDHA itself or its downstream metabolites affects the expression of cancer‐related genes. Secondly, taking into account the high heterogeneity of tumors, LDHA's biological effects and its roles in the diagnosis and therapy of tumors of different tissue origins, different pathological types, and different molecular subtypes need to be further evaluated. Finally, in addition to sensitizing traditional cytotoxic chemotherapeutics, LDHA inhibitors need to be further explored when used in conjunction with molecular targeted drugs and immunotherapy.

## CONFLICT OF INTEREST

The authors have no conflict of interests to declare.

Informed consent: Informed consent was obtained from all individual participants included in the study.
